# Machine Learning Model Based on Multiparametric MRI for Distinguishing HER2 Expression Level in Breast Cancer

**DOI:** 10.3390/curroncol33010053

**Published:** 2026-01-16

**Authors:** Yongxin Chen, Weifeng Liu, Wenjie Tang, Qingcong Kong, Siyi Chen, Shuang Liu, Liwen Pan, Yuan Guo, Xinqing Jiang

**Affiliations:** 1 The First School of Clinical Medicine, Jinan University, Guangzhou 510632, China; yongxinchen@stu2023.jnu.edu.cn; 2 Department of Radiology, Guangzhou First People’s Hospital, South China University of Technology, Guangzhou 510180, China; 3 Department of Radiology, The Third Affiliated Hospital of Sun Yat-sen University, Guangzhou 510630, China

**Keywords:** breast cancer, human epidermal growth factor receptor 2, multiparametric MRI, SHapley Additive exPlanation, machine learning

## Abstract

This study aimed to develop machine learning models based on conventional MRI features to classify HER2 expression levels in invasive breast cancer and explore their association with disease-free survival (DFS). A total of 678 patients from two centers were included, with Center 1 divided into training and internal test sets and Center 2 serving as an external test set. Random Forest models were trained to distinguish HER2-positive vs. HER2-negative (Task 1) and HER2-low vs. HER2-zero tumors (Task 2) using BI-RADS–based MRI features. SHapley Additive exPlanations were applied to rank feature importance, assist feature selection, and enhance model interpretability. DFS was analyzed using Kaplan–Meier curves and log-rank tests. In Task 1, key features included tumor size, axillary lymph nodes, fibroglandular tissue, peritumoral edema, and multifocal, achieving AUCs of 0.75 and 0.73 in the internal and external test sets, respectively. In Task 2, tumor size, peritumoral edema, and multifocal yielded AUCs of 0.73 and 0.72, respectively. Higher task-specific model scores were associated with shorter DFS in Task 1 (*p* = 0.037) and longer DFS in Task 2 (*p* = 0.046). MRI-based machine learning models can noninvasively stratify HER2 expression levels, with potential for prognostic stratification and clinical application.

## Simple Summary

Human epidermal growth factor receptor 2 (HER2) plays a crucial role in guiding treatment decisions and predicting outcomes in breast cancer. Recent advances indicate that patients with low HER2 expression may also benefit from novel targeted therapies, underscoring the need for accurate and noninvasive assessment of HER2 status. In this study, we developed machine learning models based on conventional magnetic resonance imaging (MRI) to classify different HER2 expression levels in invasive breast cancer. The models showed potential in distinguishing HER2-positive, HER2-low, and HER2-zero tumors and showed exploratory associations between model scores and survival outcomes. Using an interpretable artificial intelligence approach, we identified key MRI features related to HER2 status, such as tumor size, lymph node involvement, and peritumoral edema. These findings suggest that MRI-based machine learning may offer a noninvasive tool to support treatment planning, with possible implications for individualized risk stratification.

## 1. Introduction

Human epidermal growth factor receptor 2 (HER2) expression levels are critical in shaping treatment strategies and prognostic outcomes in invasive breast cancer. Traditionally, HER2 status has been classified into HER2-positive, treated with HER2-targeted therapies, and HER2-negative, where treatment primarily relies on hormone receptor status [1,2]. Recent studies, however, suggest a shift in this paradigm. HER2-low tumors [immunohistochemistry (IHC) score 1+ or 2+ without fluorescence in situ hybridization (FISH) amplification], which make up nearly half of invasive breast cancers and were previously grouped with HER2-negative, have shown potential for meaningful survival benefits when treated with novel antibody-drug conjugates [3,4]. As a result, accurate HER2 stratification into three categories—HER2-positive, HER2-low, and HER2-zero—has gained increasing significance in clinical practice.

HER2 status is typically evaluated using IHC and/or FISH on core needle biopsy (CNB) or surgical specimens [5,6]. However, discrepancies in HER2 expression between CNB and surgical specimens have been reported, particularly in HER2-low and HER2-zero tumors [7,8]. These inconsistencies may require repeated testing, leading to increased physical and emotional burdens for patients [6]. Thus, a non-invasive preoperative method for assessing HER2 expression levels is urgently needed.

Magnetic resonance imaging (MRI) plays an increasingly vital role in breast disease management, including diagnosis, categorization, and treatment monitoring [9,10]. Several studies have applied MRI-based radiomics to classify HER2 expression levels, achieving AUC values of 0.63 to 0.82 [11,12,13,14,15,16,17]. While radiomics shows promise, concerns remain regarding its robustness and generalizability across datasets and MRI systems [18]. Conventional MRI features, assessed by experienced radiologists using BI-RADS, have demonstrated strong performance in benign-malignant classification models [19]. Zhou et al. reported that machine learning (ML) models using conventional MRI features and Random Forest (RF) for feature selection achieved AUC values of 0.69 to 0.79 for classifying HER2 expression into three categories [20]. These findings suggest that more advanced, nonlinear feature selection methods could enhance the performance of conventional MRI models, making them comparable to radiomics-based approaches. However, the increased complexity of such methods may reduce interpretability and limit clinical trust [21,22].

SHapley Additive exPlanation (SHAP) is a game-theory-based interpretation method [23] that can be applied to a wide range of ML models, offering both local and global explanations [24]. By quantifying the marginal contribution of each feature to the model’s predictions, SHAP has the potential to provide a more refined assessment of feature importance, even when variables are not completely independent [25]. Additionally, SHAP analysis may enable more intuitive visualizations of the decision-making process, enhancing model interpretability and offering deeper insights into feature contributions [26].

Based on these findings, this study aimed to construct ML models using conventional MRI features to distinguish the HER2 triple classifications, utilize SHAP to evaluate feature contributions and improve interpretability, and explore the model’s clinical relevance through survival analysis.

## 2. Materials and Methods

### 2.1. Study Sample

**Figure 1:**
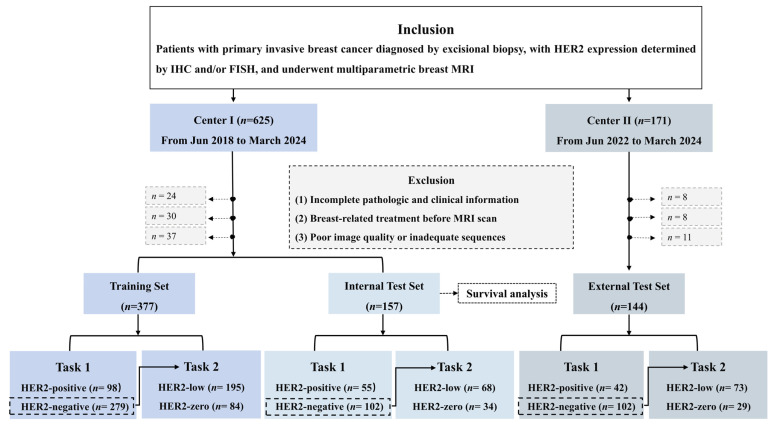
Flowchart of patient selection in this study. IHC, immunohistochemistry; FISH, fluorescent in situ hybridization; Task 1, to differentiate HER2-positive from HER2-negative (i.e., HER2-zero or HER2-low) tumors; Task 2, is performed within the HER2-negative subset from Task 1, to differentiate HER2-low from HER2-zero tumors.

This retrospective study enrolled 796 patients with invasive breast cancer (Center 1: *n* = 625; Center 2: *n* = 171), whose HER2 status was assessed by IHC and/or FISH. All patients underwent multiparametric MRI within two weeks prior to surgery, performed between June 2018 and March 2024 at Center 1, and between June 2022 and March 2024 at Center 2. A total of 118 patients were excluded due to (i) incomplete pathological or clinical data, (ii) prior breast-related treatments before MRI, or (iii) poor image quality or incomplete sequences. Ultimately, 678 patients were included in the analysis (Center 1: *n* = 534; Center 2: *n* = 144; Figure 1).

**Figure 2:**
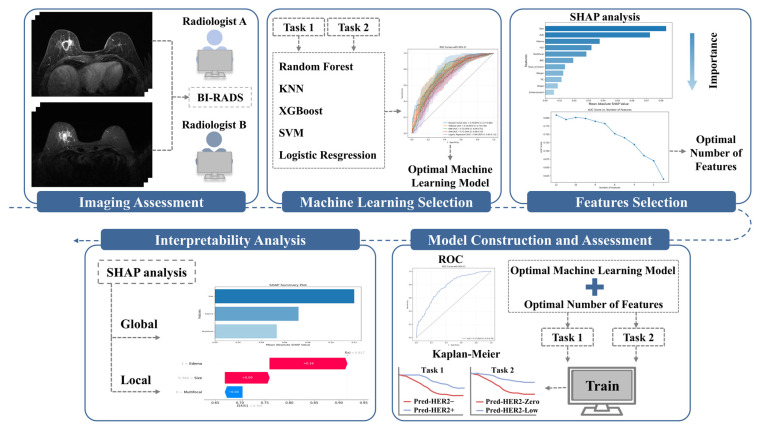
Workflow of study design. Task 1, to differentiate HER2-positive from HER2-negative (i.e., HER2-zero or HER2-low) tumors; Task 2, to differentiate HER2-low from HER2-zero tumors. In the Kaplan–Meier plots, different colored curves denote different prediction-defined subgroups, as indicated in the legend. BI-RADS, Breast Imaging Reporting and Data System; ROC, receiver operating characteristic; SHAP, SHapley Additive exPlanation; KNN, K-nearest neighbors; XGBoost, extreme gradient boosting; SVM, support vector machine; Pred-HER2–, predicted HER2-Negative; Pred-HER2+, predicted HER2-Positive; Pred-HER2-Zero, predicted HER2-Zero; Pred-HER2-Low, predicted HER2-Lowc.

This study was designed for two tasks: Task 1 aimed to distinguish HER2-positive from HER2-negative tumors, and Task 2 aimed to differentiate HER2-low from HER2-zero tumors. For each task, data from Center 1 were split by time into a training set (June 2020 to March 2024) and an internal test set (June 2018 to May 2020), which was also used for survival analysis; data from Center 2 served as an independent external test set. The study design flow is shown in Figure 2.

The present study was conducted at the same two institutions as our previous radiomics-based work on HER2 prediction [14]. Using unique patient identifiers, 445 patients overlapped between the two studies, and 233 patients were newly included in the current cohort due to the extended accrual period. While the prior study combined radiomics with conventional MRI descriptors, the present study focuses on machine-learning models based solely on conventional MRI descriptors and SHAP-assisted interpretation. All analyses in the current work were performed de novo, without reusing any radiomics features, segmentation-derived radiomics outputs, model parameters, or results from the prior publication.

### 2.2. Clinicopathologic Data Collection

Two pathologists reviewed HER2 expression levels from both centers according to the American Society of Clinical Oncology/College of American Pathologists recommendations [6]. HER2 expression was classified as HER2-zero (IHC 0), HER2-low (IHC 1+ or 2+ and FISH-negative), or HER2-positive (IHC 3+ or IHC 2+ and FISH-positive) [3]. HER2-zero and HER2-low were considered HER2-negative.

Clinical and pathological variables, including age, menopausal status, tumor location, histological type, estrogen receptor (ER) status, progesterone receptor (PR) status, and Ki67 status, was retrieved from electronic medical records. ER and PR status were considered positive when nuclear staining was observed in ≥1% of tumor cells; otherwise, results were classified as negative. Hormone receptor (HR) positivity was defined as positivity for ER and/or PR according to these criteria. Ki67 status was classified as high when >14% and low otherwise [9].

### 2.3. Breast MRI Acquisition

MRI examinations were performed on different scanners at the two institutions. At Center 1, examinations were performed on a 1.5-T system (uMR 560, United Imaging, Shanghai, China) using a 4-channel dedicated breast coil. At Center 2, MRI was performed on 3.0-T systems (DiscoveryC750 and Architect, GE Healthcare, Chicago, IL, USA) with an 8-channel breast coil. The protocol comprised T1-weighted images (T1WI), fat-suppressed T2-weighted images (T2WI), axial single-shot diffusion-weighted imaging, and dynamic contrast-enhanced (DCE) images. A pre-contrast fat-suppressed T1WI acquisition was obtained prior to DCE imaging. A gadolinium-based contrast agent (Gd-DTPA, Magnevist; Bayer HealthCare, Leverkusen, Germany) was injected intravenously at a dose of 0.2 mL/kg and a rate of 1.5 mL/s, followed by a 20 mL saline flush. Additional MRI sequence parameters are summarized in Table S1.

### 2.4. Conventional MRI Features Assessment

MRI features were independently evaluated by two radiologists blinded to HER2 expression. Features included tumor size, fibroglandular tissue (FGT) (fatty/scattered vs. heterogeneous/extremely dense), background parenchymal enhancement (BPE) (minimal/mild vs. moderate/marked), multifocal (single vs. multiple), lesion type (NST vs. ILC and other), shape (round/oval vs. irregular), margin (circumscribed vs. not circumscribed), internal enhancement (homogeneous vs. heterogeneous), enhancement curve (ascendant and/or plateau vs. washout), peritumoral edema (absent vs. present), and abnormal axillary lymph nodes (ALNs) (absent vs. present). Tumor size was defined as the maximum diameter measured on the early phase of DCE [27]. Peritumoral edema was defined as a hyperintense signal adjacent to the tumor on axial or sagittal T2WI [20]. ALNs were considered abnormal if any of the following were present [28]: absent fatty hilum, short-axis diameter > 10 mm, long-to-short-axis ratio < 2, cortical thickening, irregular margins, or asymmetry in number or size compared to the contralateral axilla. All remaining descriptors were evaluated in accordance with the BI-RADS Atlas 5th edition. For multifocal lesions, assessments were based on the largest lesion.

Tumor size was calculated as the mean of the diameters measured by the two radiologists. For other features, disagreements were resolved first through discussion between the two radiologists. If consensus could not be reached, a third radiologist made the final determination.

### 2.5. Model Construction and Evaluation

For both tasks, the modeling process consisted of three steps:

- Step 1: Five ML models were selected, including RF, support vector machine (SVM), extreme gradient boosting (XGBoost), K-nearest neighbors (K-NN), and logistic regression (LR). Before model construction, continuous variables were standardized. To mitigate class imbalance, SMOTE (Synthetic Minority Over-sampling Technique) was applied to the training data by generating synthetic samples for the minority class. Hyperparameters were optimized using a combination of grid search and manual fine-tuning. Each model was validated using 10-fold cross-validation, and the model with the highest mean AUC was selected for the next step.
- Step 2: Feature selection was based on the contribution of each feature in the selected ML model, ranking them by importance [24]. Features were progressively removed in ascending order of importance, with the AUC recalculated at each step. The process was halted when the AUC reduction became statistically significant compared to the model with all features, as determined by the DeLong test [24]. The number of features at this point was finalized for the model, balancing predictive performance and feature reduction.
- Step 3: Using the selected features from Step 2, the final ML model was developed and validated through 10-fold cross-validation. Performance was evaluated using several commonly applied metrics, including the area under the receiver operating characteristic (ROC) curve, accuracy (ACC), specificity (SPE), sensitivity (SEN), positive predictive value (PPV), and negative predictive value (NPV).

### 2.6. SHAP-Based Interpretability Analysis

The SHAP package in Python (v3.12.3) was used to provide both global and local interpretations for the ML models in both tasks. Global interpretation aimed to assign importance values to each model feature. This was achieved by calculating SHAP values for each feature and generating SHAP summary plots, which display the mean absolute SHAP values across all patients to illustrate the overall contribution of each feature to the model. In addition, swarm plots are used to show the correlation between each feature and the model predictions. Local interpretation focused on understanding individual predictions by constructing waterfall plots, which highlight the features that contributed most significantly to the model’s predicted probability for a specific patient, emphasizing their impact on individual outcomes.

### 2.7. Survival Analysis

Survival analysis was performed in the internal test set to explore the association between task-specific model outputs and disease-free survival (DFS). DFS was defined as the time interval from the date of surgery to the occurrence of tumor recurrence, distant metastasis, or death [29]. Follow-up and recurrence data for the internal test set (June 2018 to May 2020) were obtained from electronic medical records, with 30 June 2023 as the data cut-off date. Consequently, follow-up duration varied among patients based on their date of surgery. Patients who did not experience recurrence or were lost to follow-up by the last follow-up date were treated as censored observations.

The model-predicted probabilities were used as task-specific model scores. The Task 1 model score was defined as the predicted probability of HER2-positive status (Task 1: HER2-positive vs. HER2-negative), and the Task 2 model score was defined as the predicted probability of HER2-low status within the pathologically HER2-negative subset (Task 2: HER2-low vs. HER2-zero). For each task, an optimal cut-off value was determined using the Youden index and used to stratify patients into high- and low-score groups. DFS was compared using Kaplan–Meier curves with the log-rank test. Associations between the model score and DFS were evaluated using univariable and multivariable Cox proportional hazards models, adjusted for age, menopausal status, tumor location, histologic type, ER status, PR status, and Ki-67.

### 2.8. Statistical Analysis

Analysis was conducted using Python v3.12.3 (https://www.python.org) and SPSS statistical software v26.0 (https://www.ibm.com/spss, accessed on 10 December 2025). Continuous variables were compared using the *t*-test or Mann–Whitney U test, while categorical variables were analyzed using the Chi-square test or Fisher’s exact test. For inter-reader agreement, the intraclass correlation coefficient (ICC) was used for continuous variables, categorized as poor (<0.75), good (0.75–0.90), or excellent (>0.90) [30]. Categorical variables were evaluated using the kappa coefficient, classified as poor (<0.00), slight (0.00–0.20), fair (0.21–0.40), moderate (0.41–0.60), substantial (0.61–0.80), or almost perfect (0.81–1.00) [31]. Model performance was evaluated using metrics such as the AUC, ACC, SEN, SPE, PPV, and NPV. The DeLong test was employed to compare differences in AUCs as features were progressively reduced during model construction. Statistical significance was defined as a two-tailed *p*-value of < 0.05.

## 3. Results

### 3.1. Patients

A total of 678 patients were included in the study, comprising 377 in the training set (HER2-zero: 84 [22.3%], HER2-low: 195 [51.7%], HER2-positive: 98 [26.0%]), 157 in the internal test set (HER2-zero: 34 [21.7%], HER2-low: 68 [43.3%], HER2-positive: 55 [35.0%]), and 144 in the external test set (HER2-zero: 29 [20.1%], HER2-low: 73 [50.7%], HER2-positive: 42 [29.2%]). Clinicopathological characteristics did not differ significantly across the three datasets (*p* > 0.05) (Table 1). The distribution of receptor-defined subgroups is summarized in Table S2.

Table S3 presents inter-reader agreement for conventional MRI features. Tumor size demonstrated excellent agreement (ICC = 0.93), while categorical features showed almost perfect agreement, with kappa values ranging from 0.80 to 0.88.

Table 2 and Table 3 summarize the univariable analysis of conventional MRI features for Task 1 and Task 2. For Task 1, tumor size, peritumoral edema, and abnormal ALNs differed significantly between HER2-positive and HER2-negative tumors across the training, internal test, and external test sets (all *p* < 0.05). By contrast, in Task 2, none of the conventional MRI features showed a statistically significant difference between HER2-low and HER2-zero tumors in any sets (all *p* > 0.05).

### 3.2. Model Construction

Five ML models were evaluated for each task to identify the optimal approach. In Task 1, 10-fold cross-validation showed that RF achieved the highest mean AUC at 0.81 (95% CI: 0.78–0.83), while LR had the lowest mean AUC of 0.63 (95% CI: 0.58–0.69). Similarly, in Task 2, RF yielded the highest mean AUC of 0.77 (95% CI: 0.73–0.81), whereas LR yielded the lowest mean AUC of 0.64 (95% CI: 0.61–0.68) (Figure S1; Tables S4 and S5).

**Figure 3:**
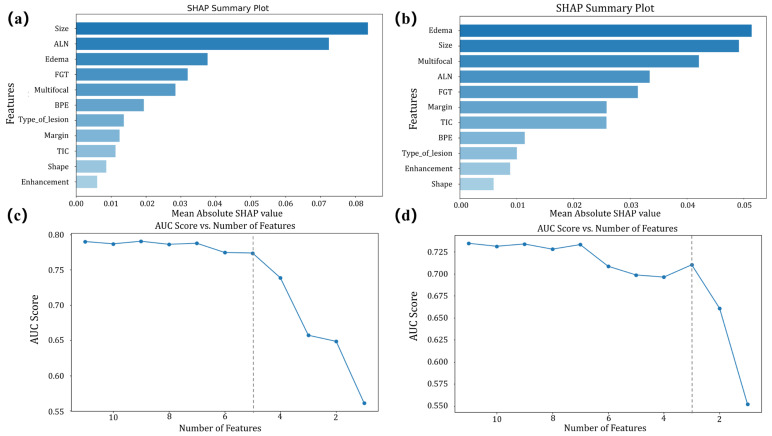
SHAP summary plots for initial feature importance and AUC changes with feature reduction in two tasks. In Task 1, (**a**) shows the initial ranking of all features by SHAP values from the model using the full feature set, indicating their contribution to the model. (**c**) presents the AUC line plot as features are progressively removed from the full set according to their importance, with the gray dashed line indicating the final number of features used. In Task 2, (**b**) shows the initial feature importance ranking for the full-feature model, and (**d**) illustrates how AUC changes as features are reduced from the full set, with the gray dashed line indicating the final number of features selected.

SHAP analysis was used to visualize and rank feature contributions in the RF model (Figure 3a,b). Features were progressively removed in ascending order of importance, and the corresponding AUC was calculated (Figure 3c,d). In Task 1, the model with 5 features (tumor size, abnormal ALNs, peritumoral edema, FGT, and multifocal) demonstrated similar performance to the model with 11 features (AUC: 0.77 vs. 0.79, *p* = 0.51). In Task 2, the model with 3 features (peritumoral edema, tumor size, and multifocal) performed similarly to the model with 11 features (AUC: 0.71 vs. 0.74, *p* = 0.36). The DeLong test results are provided in Tables S6 and S7.

### 3.3. Model Performance

**Figure 4:**
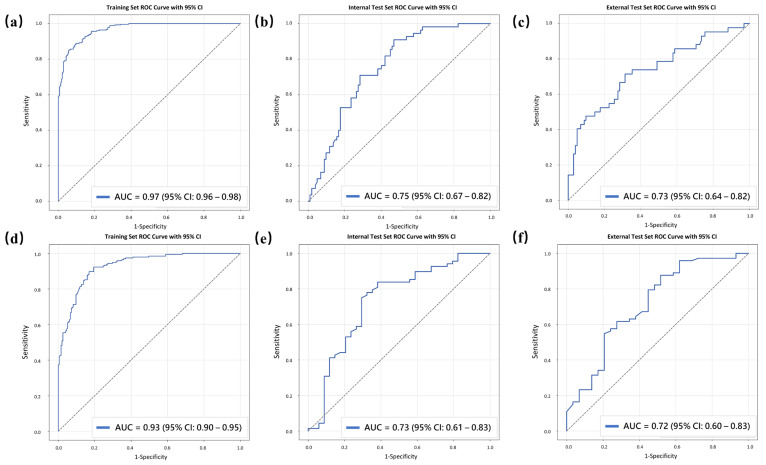
ROC curves for the final RF model for two tasks across the three sets. For Task 1, the final RF model achieved AUCs of 0.97 in the training set (**a**), 0.75 in the internal test set (**b**), and 0.73 in the external test set (**c**). For Task 2, the final RF model achieved AUCs of 0.93 in the training set (**d**), 0.73 in the internal test set (**e**), and 0.72 in the external test set (**f**). The gray dashed diagonal line represents the reference line for random classification.

For Task 1, the final RF model yielded AUCs of 0.97 (95% CI: 0.96–0.98) in the training set, 0.75 (95% CI: 0.67–0.82) in the internal test set, and 0.73 (95% CI: 0.64–0.82) in the external test set (Figure 4a–c). For Task 2, the corresponding AUCs were 0.93 (95% CI: 0.90–0.95), 0.73 (95% CI: 0.61–0.83), and 0.72 (95% CI: 0.60–0.83), respectively (Figure 4d–f). Detailed performance metrics for both tasks across the three datasets are summarized in Table 4. Confusion matrices for both tasks in the internal and external test sets are also provided in Figure S2.

### 3.4. Interpretability Analysis

**Figure 5:**
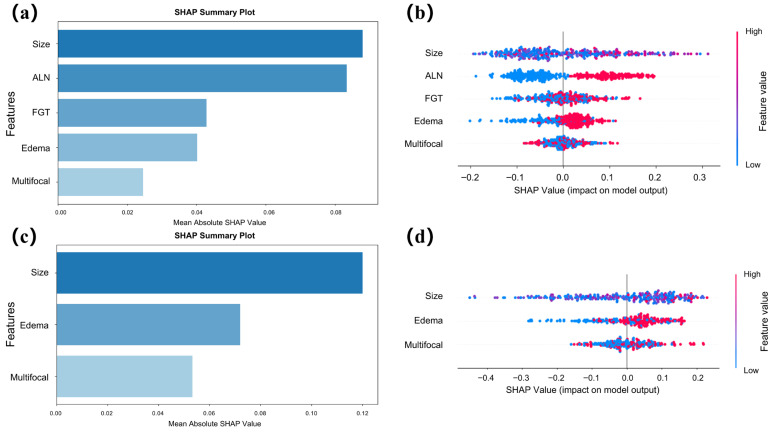
SHAP summary plots and swarm plots for global explanations of the final models in Task 1 (**a**,**b**) and Task 2 (**c**,**d**). In each task, the SHAP summary plot (**a**,**c**) shows feature importance for the final model trained on the selected feature subset, where longer strips represent higher mean absolute SHAP values and greater feature contributions. In the SHAP swarm plot (**b**,**d**) for each feature, single points represent distinct patients; points are distributed horizontally along x-axis in accordance with their SHAP values. Points are stacked vertically in areas of high density of SHAP values. Red points indicate variables that are relatively high in actual value for specific instance, whereas blue points indicate variables that are relatively low in actual value.

SHAP summary and swarm plots were used to provide global explanations of the models for both tasks (Figure 5). In Task 1, tumor size and abnormal ALNs ranked as the top contributors, as shown by the long bars at the top of the summary plot, indicating their high mean impact on the model’s predictions (Figure 5a). The swarm plot further revealed that larger tumor sizes and the presence of abnormal ALNs were more strongly associated with HER2-positive tumors (Figure 5b). In Task 2, tumor size remained the most influential feature, with the swarm plot demonstrating a stronger association between larger tumor size and HER2-low tumors (Figure 5c,d). Additionally, peritumoral edema was highly correlated with HER2-positive tumors in Task 1 but showed a greater association with HER2-low tumors in Task 2.

**Figure 6:**
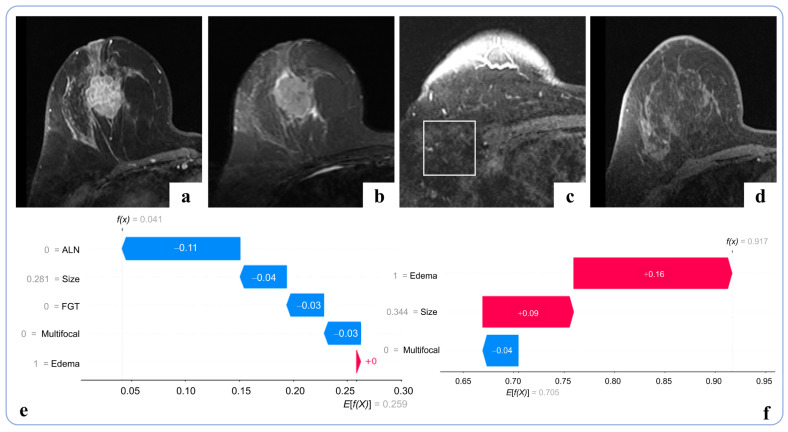
Patient-specific local interpretation of the models for both tasks visualized using SHAP waterfall plots. The case involves a patient with invasive breast cancer with HER2-low expression. (**a**) Depicts an axial DCE-MRI image highlighting a single lesion; (**b**) shows an axial T2WI revealing the presence of peritumoral edema; (**c**) axial DCE-MRI image of the axillary region with a rectangular annotation highlighting the area assessed. No abnormal lymph nodes are identified; and (**d**) demonstrates scattered FGT. The Task 1 (**e**) model predicts a 4.1% probability of HER2 positivity for this patient, while the Task 2 (**f**) model outputs a 91.7% probability of HER2-low expression. In (**e**,**f**), red bars indicate positive contributions to the model output, whereas blue bars indicate negative contributions.

SHAP waterfall plots were used to provide local explanations based on individual predictions (Figure 6 and Figures S3 and S4). In Figure 6, for Task 1, most features reduced the likelihood of HER2-positive classification for this patient, while peritumoral edema showed an opposite trend (predicted probability: 4.1%). In contrast, for Task 2, the presence of peritumoral edema contributed the most to the predicted HER2-low probability of 91.7% for the same patient.

### 3.5. Task-Specific Survival Analysis

**Figure 7:**
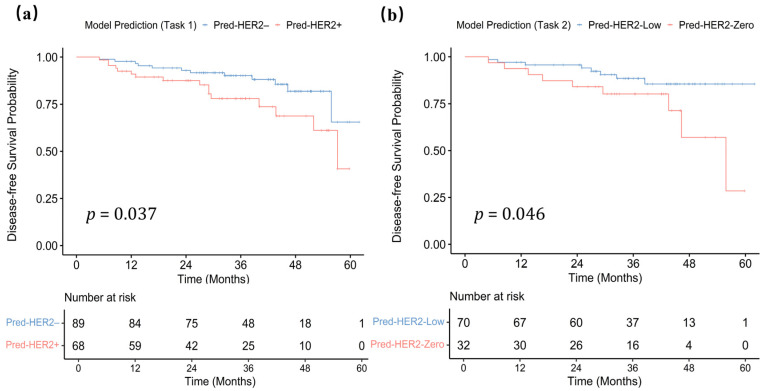
Kaplan–Meier survival curves of disease-free survival (DFS) stratified by task-specific model predictions in the internal test set. (**a**) For Task 1 (HER2-positive vs. HER2-negative), patients with a higher Task 1 model score (predicted HER2-positive) had shorter DFS (*p* = 0.037). (**b**) For Task 2 (HER2-low vs. HER2-zero) within the pathologically HER2-negative subset, patients with a higher Task 2 model score (predicted HER2-low) had longer DFS (*p* = 0.046). Pred-HER2–, predicted HER2-Negative; Pred-HER2+, predicted HER2-Positive; Pred-HER2-Low, predicted HER2-Low; Pred-HER2-Zero, predicted HER2-Zero.

Survival analysis was conducted in the internal test set. In Task 1, the median follow-up time was 34.0 months (IQR: 22.5–45.2 months). During the follow-up period, 28 patients (17.83%) experienced recurrence. Patients were stratified into low vs. high Task 1 model score groups using a cutoff value of 0.420. Kaplan–Meier survival curve demonstrated that a high Task 1 model score (predicted HER2-positive) was significantly associated with shorter DFS (*p* = 0.037; Figure 7a). In Cox regression analysis, the Task 1 model score was significantly associated with DFS in univariable analysis, and after adjustment for clinicopathologic factors in the multivariable model, a high Task 1 model score remained an independent predictor of poorer DFS (HR 2.26, 95% CI 1.06–4.84; *p* = 0.035; Table S8).

In Task 2, the median follow-up time was 36.0 months (IQR: 27.7–45.3 months). During the follow-up period, 17 patients (16.67%) experienced recurrence. Patients were stratified into low vs. high Task 2 model score groups using a cutoff value of 0.496. Kaplan–Meier survival curve showed that a high Task 2 model score (predicted HER2-low) was significantly associated with longer DFS (*p* = 0.046; Figure 7b). However, in Cox analyses adjusted for clinicopathologic variables, the association between the Task 2 model score and DFS did not reach statistical significance, showing only a borderline trend (HR 2.423, 95% CI 0.930–6.317; *p* = 0.070; Table S9).

## 4. Discussion

This study utilized ML models based on conventional MRI features combined with SHAP to visualize feature importance rankings, aiding in feature selection for distinguishing the three levels of HER2 expression. The results demonstrated that this approach achieved AUCs of 0.75 and 0.73 in distinguishing HER2-positive from HER2-negative tumors in the internal and external test sets, respectively, and AUCs of 0.73 and 0.72 in distinguishing HER2-low from HER2-zero tumors. In exploratory survival analyses, DFS differed between the model-defined groups, suggesting that the task-specific MRI-derived model scores may be associated with clinical outcomes.

### 4.1. Comparison with Prior Studies

Recent studies on HER2 triple classification primarily focus on the application of MRI radiomics [12,15,16,32]. Several studies have reported robust performance in distinguishing HER2-positive from HER2-negative tumors. Zheng et al. [9] developed a radiomics model using T2WI, DCE, diffusion-weighted imaging, and apparent diffusion coefficient (ADC) images, achieving an AUC of 0.725; Bian et al. [13] constructed a combined model using T1WI contrast-enhanced and ADC imaging features, achieving an AUC of 0.76. Luo et al. used T2WI and DCE-MRI radiomics in a machine learning model and achieved an AUC of 0.777 [17]. For the more clinically challenging task of distinguishing HER2-low from HER2-zero tumors, these studies also showed similar performance, with AUC values ranging from 0.71 to 0.77 [13,17]. Overall, these radiomics models achieved performance that was in a comparable range, with some reporting slightly higher AUCs. However, radiomics-based performance may be more sensitive to differences in datasets, MRI systems, and preprocessing pipelines, as it relies on standardized image acquisition and feature-extraction procedures, which can limit reproducibility and routine clinical implementation [18].

In contrast, studies using conventional MRI features for HER2 classification remain limited [20,33]. Zhou et al. developed ML models based on BI-RADS MRI features and reported an AUC of 0.79 (KNN model) for distinguishing HER2-zero from non-zero tumors, and an AUC of 0.69 (DT model) for differentiating HER2-low from HER2-positive tumors [20]. Their overall performance was slightly better than ours, which may be related to differences in study design and the feature selection strategies. Despite these differences, their findings, together with ours, underscore the potential of ML methods based on conventional MRI features for HER2 classification, as these methods are capable of capturing complex, non-linear relationships between imaging features and HER2 status.

### 4.2. Interpretability and Feature Relevance

Previous studies have shown that in HER2-positive tumors, abnormal ALNs and larger tumor size are commonly associated with more aggressive biological features, while peritumoral edema indicates vascular invasion around the tumor, reflecting the invasive characteristics of HER2-positive tumors [9,13,20]. These findings are consistent with those of our study. However, when distinguishing HER2-low from HER2-zero tumors, we did not observe significant differences in feature distribution, which is consistent with previous findings [14]. Due to the lack of distribution differences, traditional univariable analysis failed to effectively select features, making it challenging to construct an effective classification model. In contrast, our study utilized SHAP to visualize feature contributions during the ML model construction process, aiding feature selection. This approach demonstrated good performance, highlighting the potential of SHAP-based interpretability analysis for this specific task. Hu et al. applied a similar SHAP-assisted method for feature selection and demonstrated that it achieved consistent performance across internal and external validations [24]. By comparing the performance of different feature subsets during model construction, this approach retained the most meaningful features, reduced model complexity, and maintained good classification accuracy.

In the SHAP analysis, tumor size and abnormal ALNs were identified as the most important features for distinguishing HER2-positive from HER2-negative tumors. These findings are consistent with our previous feature distributions analysis and align with the results from traditional univariable analysis, further confirming the importance of these conventional MRI features in HER2 classification. When distinguishing HER2-low from HER2-zero tumors, tumor size remained the most important feature in SHAP global analysis, although previous studies have not reached a consensus on the distribution differences in tumor size between HER2-low and HER2-zero tumors [34,35,36]. Interestingly, peritumoral edema, as a binary variable, played a significant role in both tasks. In Task 1, the presence of peritumoral edema was strongly associated with HER2-positive tumors, while in Task 2, it showed a stronger association with HER2-low tumors. This finding aligns with those of Zhou et al., who observed that the incidence of peritumoral edema increased with higher HER2 scores [20]. Although no significant differences in the distribution of peritumoral edema were observed between HER2-low and HER2-zero tumors, SHAP analysis uncovered a complex relationship between peritumoral edema and HER2-negative tumors. By evaluating the marginal effects of features, SHAP analysis provided a more nuanced assessment of feature contributions, facilitating model optimization and deeper insights into tumor biology.

### 4.3. Exploratory Survival Analysis

Survival analysis in the internal test set suggested that the task-specific model outputs derived from conventional MRI features may carry prognostic information. In Task 1, patients predicted as HER2-positive had shorter DFS, and this association remained significant after adjustment for clinicopathologic factors, indicating that the imaging-based HER2 phenotype captured by the model may be biologically relevant and correlates with the aggressive behavior of HER2-positive tumors. Within the HER2-negative population (Task 2), predicted HER2-low patients were associated with longer DFS on Kaplan–Meier analysis, which is consistent with previous reports suggesting that HER2-low tumors may have a more favorable prognosis than HER2-zero tumors [11,37]. However, this association did not remain statistically significant after multivariable Cox adjustment, possibly reflecting the limited number of events and the complex interplay between HER2 expression and other biological and treatment-related factors. Therefore, these survival findings should be interpreted with caution and require validation in future studies with larger sample sizes.

### 4.4. Limitations

This study has several limitations. Firstly, due to the retrospective nature of this study, there may be an unavoidable sample selection bias, although we have conducted a comparison of the included populations of the different datasets. Therefore, future multi-center, prospective studies are required to validate the results of this study. Second, the evaluation of conventional MRI features was performed by radiologists trained at the same center. However, the applicability of this model when evaluated by radiologists from other centers or with different levels of experience remains uncertain. Therefore, further validation involving radiologists from diverse clinical settings and with varying years of experience is needed to assess the model’s generalizability.

## 5. Conclusions

The ML model based on conventional MRI features, assisted by SHAP analysis, can help distinguish different levels of HER2 expression and may contribute to prognostic stratification, offering valuable insights for personalized patient management.

## Figures and Tables

**Table 1:** Clinicopathological characteristics of patients from three datasets.

Feature	Training Set (*n* = 377)	Internal Test Set (*n* = 157)	External Test Set (*n* = 144)	*p* Value
Age (y), mean ± SD	54.69 ± 11.12	54.85 ± 10.54	52.76 ± 10.18	0.811
Menopausal status				0.165
Yes	248 (65.78)	105 (66.88)	83 (57.64)	
No	129 (34.22)	52 (33.12)	61 (42.36)	
Location				0.377
Left	203 (53.85)	77 (49.04)	82 (56.94)	
Right	174 (46.15)	80 (50.96)	62 (43.06)	
Histologic type				0.135
NST	350 (92.84)	145 (92.36)	126 (87.50)	
ILC and other	27 (7.16)	12 (7.64)	18 (12.50)	
HER2 expression				0.286
HER2-positive	98 (25.99)	55 (35.03)	42 (29.17)	
HER2-low	195 (51.72)	68 (43.31)	73 (50.69)	
HER2-zero	84 (22.28)	34 (21.66)	29 (20.14)	
ER				0.932
Positive	296 (78.51)	121 (77.07)	112 (77.78)	
Negative	81 (21.49)	36 (22.93)	32 (22.22)	
PR				0.539
Positive	270 (71.62)	111 (70.70)	96 (66.67)	
Negative	107 (28.38)	46 (29.30)	48 (33.33)	
Ki67				0.489
≤14%	294 (77.98)	115 (73.25)	109 (75.69)	
>14%	83 (22.02)	42 (26.75)	35 (24.31)	

Unless otherwise indicated, data expressed as count, with percentage in parentheses. NST, invasive carcinoma of no special type; ILC, invasive lobular carcinoma; HER2, human epidermal growth factor receptor 2; ER, estrogen receptor; PR, progesterone receptor.

**Table 2:** Univariable analysis of conventional MRI features in training, internal test, and external test sets for Task 1.

Feature	Training Set (*n* = 377)			Internal Test Set (*n* = 157)			External Test Set (*n* = 144)		
	HER2- Positive (*n* = 98)	HER2- Negative (*n* = 279)	*p*	HER2- Positive (*n* = 55)	HER2- Negative (*n* = 102)	*p*	HER2- Positive (*n* = 42)	HER2- Negative (*n* = 102)	*p*
Tumor size (mm)	33.44 ± 17.40	28.99 ± 18.07	0.03	29.62 ± 12.61	24.42 ± 11.91	0.01	31.02 ± 12.11	25.59 ± 12.63	0.02
FGT			0.34			0.11			0.83
Fatty/scattered	43 (43.88)	140 (50.18)		19 (34.55)	50 (49.02)		17 (40.48)	45 (44.12)	
Heterogeneous/extremely dense	55 (56.12)	139 (49.82)		36 (65.45)	52 (50.98)		25 (59.52)	57 (55.88)	
BPE			>0.99			>0.99			0.14
Minimal/mild	80 (81.63)	228 (81.72)		48 (87.27)	89 (87.25)		36 (85.71)	74 (72.55)	
Moderate/marked	18 (18.37)	51 (18.28)		7 (12.73)	13 (12.75)		6 (14.29)	28 (27.45)	
Multifocal			0.20			0.10			0.12
Single	50 (51.02)	165 (59.14)		28 (50.91)	67 (65.69)		22 (52.38)	69 (67.65)	
Multiple	48 (48.98)	114 (40.86)		27 (49.09)	35 (34.31)		20 (47.62)	33 (32.35)	
Tumor shape			0.16			>0.99 ^a^			0.72 ^a^
Round/oval	2 (2.04)	18 (6.45)		1 (1.82)	2 (1.96)		2 (4.76)	8 (7.84)	
Irregular	96 (97.96)	261 (93.55)		54 (98.18)	100 (98.04)		40 (95.24)	94 (92.16)	
Tumor margin			0.07			0.34			0.33
Circumscribed	7 (7.14)	42 (15.05)		4 (7.27)	14 (13.73)		3 (7.14)	15 (14.71)	
Not circumscribed	91 (92.86)	237 (84.95)		51 (92.73)	88 (86.27)		39 (92.86)	87 (85.29)	
Mass internal enhancement			0.58			>0.99 ^a^			0.23 ^a^
Homogeneous	7 (7.14)	27 (9.68)		3 (5.45)	7 (6.86)		2 (4.76)	13 (12.75)	
Heterogeneous	91 (92.86)	252 (90.32)		52 (94.55)	95 (93.14)		40 (95.24)	89 (87.25)	
Enhancement curve			0.75			0.25 ^a^			>0.99
Ascendant and/or plateau	186 (87.76)	250 (89.61)		48 (87.27)	95 (93.14)		36 (85.71)	87 (85.29)	
Washout	12 (12.24)	29 (10.39)		7 (12.73)	7 (6.86)		6 (14.29)	15 (14.71)	
Nonmass enhancement			0.15			0.48			0.16
Absent	70 (71.43)	221 (79.21)		39 (70.91)	79 (77.45)		31 (73.81)	87 (85.29)	
Present	28 (28.57)	58 (20.79)		16 (29.09)	23 (22.55)		11 (26.19)	15 (14.71)	
Peritumoral edema			0.02			<0.001			0.002
Absent	23 (23.47)	104 (37.28)		7 (12.73)	45 (44.12)		7 (16.67)	46 (45.10)	
Present	75 (76.53)	175 (62.72)		48 (87.27)	57 (55.88)		35 (83.33)	56 (54.90)	
Abnormal ALNs			<0.001			<0.001			<0.001
Absent	37 (37.76)	181 (64.87)		14 (25.45)	57 (55.88)		15 (35.71)	74 (72.55)	
Present	61 (62.24)	98 (35.13)		41 (74.55)	45 (44.12)		27 (64.29)	28 (27.45)	

^a^ Calculated by Fisher’s exact test. FGT, fibroglandular tissue; BPE, background parenchymal enhancement; ALN, axillary lymph node.

**Table 3:** Univariable analysis of conventional MRI features in training, internal test, and external test sets for Task 2.

Feature	Training Set (*n* = 279)			Internal Test Set (*n* = 102)			External Test Set (*n* = 102)		
	HER2- Low (*n* = 195)	HER2- Zero (*n* = 84)	*p*	HER2- Low (*n* = 68)	HER2- Zero (*n* = 34)	*p*	HER2- Low (*n* = 73)	HER2- Zero (*n* = 29)	*p*
Tumor size (mm)	30.01 ± 19.10	26.62 ± 15.24	0.15	24.14 ± 11.34	24.98 ± 13.14	0.74	26.26 ± 13.24	23.90 ± 10.97	0.40
FGT			0.72			0.94			0.43
Fatty/scattered	96 (49.23)	44 (52.38)		34 (50.00)	16 (47.06)		34 (46.58)	11 (37.93)	
Heterogeneous/extremely dense	99 (50.77)	40 (47.62)		34 (50.00)	18 (52.94)		39 (53.42)	18 (62.07)	
BPE			0.96			0.76			0.99
Minimal/mild	160 (82.95)	68 (80.95)		60 (88.24)	29 (85.29)		53 (72.60)	21 (72.41)	
Moderate/marked	35 (17.95)	16 (19.05)		8 (11.76)	5 (14.71)		20 (27.40)	8 (27.59)	
Multifocal			0.31			0.34			0.11
Single	111 (56.92)	54 (64.29)		42 (61.76)	25 (73.53)		47 (64.38)	23 (79.31)	
Multiple	84 (43.08)	30 (35.71)		26 (38.24)	9 (26.47)		26 (35.62)	6 (20.69)	
Tumor shape			>0.99			0.55 ^a^			0.22 ^a^
Round/oval	13 (6.67)	5 (5.95)		2 (2.94)	0 (0.00)		4 (5.48)	4 (13.79)	
Irregular	182 (93.33)	79 (94.05)		66 (97.06)	34 (100.00)		69 (94.52)	25 (86.21)	
Tumor margin			0.16			>0.99 ^a^			0.76 ^a^
Circumscribed	25 (12.82)	17 (20.24)		9 (14.71)	5 (14.71)		10 (13.70)	5 (17.24)	
Not circumscribed	170 (87.18)	67 (79.76)		59 (85.76)	29 (85.29)		63 (86.30)	24 (82.76)	
Mass internal enhancement			>0.99			0.42 ^a^			>0.99 ^a^
Homogeneous	19 (9.74)	8 (9.52)		6 (8.82)	1 (2.94)		9 (12.33)	3 (10.34)	
Heterogeneous	176 (90.26)	76 (90.48)		62 (91.18)	33 (97.06)		64 (87.67)	26 (89.66)	
Enhancement curve			0.17			>0.99 ^a^			0.06 ^a^
Ascendant and/or plateau	171 (87.69)	79 (94.05)		63 (92.65)	32 (94.12)		59 (80.82)	28 (96.55)	
Washout	24 (12.31)	5 (5.95)		5 (7.35)	2 (5.88)		14 (19.18)	1 (3.45)	
Nonmass enhancement			0.99			0.68			0.22 ^a^
Absent	155 (79.49)	66 (78.57)		54 (79.41)	25 (73.53)		60 (82.19)	27 (93.10)	
Present	40 (20.51)	18 (21.43)		14 (20.59)	9 (26.47)		13 (17.81)	2 (6.90)	
Peritumoral edema			0.052			0.83			0.08
Absent	65 (33.33)	39 (46.43)		29 (42.65)	16 (47.06)		29 (39.73)	17 (58.62)	
Present	130 (66.67)	45 (53.57)		39 (57.35)	18 (52.94)		44 (60.27)	12 (41.38)	
Abnormal ALNs			0.27			0.29			0.15
Absent	122 (62.56)	59 (70.24)		41 (60.29)	16 (47.06)		50 (68.49)	24 (82.76)	
Present	73 (37.44)	25 (29.76)		27 (39.71)	18 (52.94)		23 (31.51)	5 (17.24)	

^a^ Calculated by Fisher’s exact test. FGT, fibroglandular tissue; BPE, background parenchymal enhancement; ALN, axillary lymph node.

**Table 4:** Performance of models for two tasks across three datasets.

Task 1, Sets	AUC (95% CI)	ACC	SEN	SPE	PPV	NPV
Training set	0.97 (0.96–0.98)	89.1	90.0	88.2	88.4	89.8
Internal test set	0.75 (0.67–0.82)	71.3	70.9	71.6	57.4	82.0
External test set	0.73 (0.64–0.82)	68.8	66.7	69.6	47.5	83.5
Task 2, Sets						
Training set	0.93 (0.90–0.95)	85.4	86.7	84.1	84.5	86.3
Internal test set	0.73 (0.61–0.83)	76.5	83.8	61.8	81.4	65.6
External test set	0.72 (0.60–0.83)	71.6	75.3	62.1	83.3	50.0

AUC, area under the curve; CI, confidence interval; ACC, accuracy; SEN, sensitivity; SPE, specificity; PPV, positive predictive value; NPV, negative predictive value.

## Data Availability

The data presented in this study are available on request from the corresponding author.

## Supplementary Materials

The following supporting information can be downloaded at: https://www.mdpi.com/article/10.3390/curroncol33010053/s1, Table S1. MRI sequences and parameters at two centers. Table S2. Distribution of HER2–hormone receptor molecular subgroups across the training, internal test, and external test sets. Table S3. Interreader agreement for conventional MRI features. Table S4. The performance of five ML models in Task 1. Table S5. The performance of five ML models in Task 2. Table S6. AUC of models with different number of features and their comparison in Task 1. Table S7. AUC of models with different number of features and their comparison in Task 2. Table S8. Univariable and multivariable Cox regression analyses of disease-free survival for Task 1 in the internal test set. Table S9. Univariable and multivariable Cox regression analyses of disease-free survival for Task 2 in the internal test set. Figure S1. ROC curves for the five machine learning models in Task 1 (a) and Task 2 (b). Figure S2. Confusion matrices for model performance on Task 1 and Task 2. Figure S3. Patient-specific local interpretation of the models for both tasks visualized using SHAP waterfall plots. Figure S4. Patient-specific local interpretation of the models for both tasks visualized using SHAP waterfall plots.

## Abbreviations

SHAP	SHapley Additive exPlanation
ML	machine learning
cMRI	conventional MRI
AUC	area under the curve
HER2	human epidermal growth factor receptor 2
IHC	immunohistochemistry
FISH	fluorescence in situ hybridization
CNB	core needle biopsy
MRI	magnetic resonance imaging
ER	estrogen receptor
PR	progesterone receptor
T1WI	T1-weighted images
T2WI	T2-weighted images
DCE	dynamic contrast-enhanced
FGT	fibroglandular tissue
BPE	background parenchymal enhancement
ALNs	axillary lymph nodes
RF	Random Forest
SVM	Support Vector Machine
XGBoost	eXtreme Gradient Boosting
K-NN	K-Nearest Neighbors
LR	Logistic Regression
ROC	the receiver operating characteristic
ACC	accuracy
SPE	specificity
SEN PPV NPV	sensitivity positive predictive value negative predictive value
DFS	disease-free survival
ICC	intraclass correlation coefficient
