# Supplementary material for: Machine Learning Model Based on Multiparametric MRI for Distinguishing HER2 Expression Level in Breast Cancer
Source: Curr Oncol. 2026 Jan 16;33(1):53. doi: 10.3390/curroncol33010053 (PMC12839683; doi:10.3390/curroncol33010053)
Supplement: Supplementary file 1 [file curroncol-33-00053-s001.zip › curroncol-3957076-supplementary.pdf]

Table S1. MRI sequences and parameters at two centers.

Scanner, Sequence	TR (ms)	TE (ms)	FOV (mm^2)	Slice Thickness (mm)	Acquisition Time (sec)
Center 1					
United Imaging 1.5 T (uMR 560)					
T2WI	3800	42.7	328×350	4.0	126
DCE	5.1	2.1	320×320	2.4	394
Center 2					
GE 3 T (Discovery 750)					
T2WI	4968	85.5	320×320	4.0	235
DCE	3.8	1.6	360×360	1.4	737
GE 3 T (Architect)					
T2WI	4831	86	360×360	4.0	275
DCE	6.5	2.9	320×320	0.6	405

All sequences used fat suppression. T2WI, T2-weighted images; DCE, dynamic contrast-enhanced. Images.

Table S2. Distribution of HER2–hormone receptor molecular subgroups across the training, internal test, and external test sets.

Molecular Subgroups	Training Set (n=377)	Internal Test Set (n=157)	External Test Set (n=144)
HER2-positive/HR+	65 (17.24)	35 (22.29)	28 (19.44)
HER2-positive/HR–	33 (8.75)	20 (12.74)	14 (9.72)
HER2-low/HR+	174 (46.15)	65 (41.40)	62 (43.06)
HER2-low/HR–	21 (5.57)	3 (1.91)	11 (7.64)
HER2-zero/HR+	65 (17.24)	25 (15.92)	24 (16.67)
HER2-zero/HR–	19 (5.04)	9 (5.73)	5 (3.47)

HR, hormone receptor.

Table S3. Interreader agreement for conventional MRI features.

Feature	Agreement (95% CI)[a]
Tumor size	0.93 (0.86-0.97)
Fibroglandular tissue	0.88 (0.84-0.92)
Background parenchymal enhancement	0.86 (0.80-0.92)
Multifocal	0.86 (0.82-0.91)
Tumor shape	0.82 (0.70-0.93)
Tumor margin	0.80 (0.73-0.88)
Mass internal enhancement	0.83 (0.75-0.91)
Enhancement curve	0.85 (0.78-0.92)
Nonmass enhancement	0.86 (0.81-0.91)
Peritumoral edema	0.81 (0.75-0.86)
Abnormal ALNs	0.83 (0.78-0.88)

[a]Expressed as intraclass correlation coefficient for tumor size and as kappa coefficient for remaining variables. ALNs, axillary lymph nodes.

Table S4. The performance of five ML models in Task 1.

Model	AUC (95% CI)	ACC	SEN	SPE	PPV	NPV
RF	0.81 (0.78-0.83)	0.75	0.78	0.71	0.73	0.77
XGBoost	0.75 (0.73-0.78)	0.69	0.71	0.66	0.68	0.70
KNN	0.74 (0.70-0.78)	0.67	0.80	0.54	0.63	0.73
SVM	0.66 (0.62-0.71)	0.63	0.59	0.67	0.64	0.62
LR	0.63 (0.58-0.69)	0.62	0.60	0.63	0.62	0.62

AUC, area under the curve; CI, confidential interval; RF, Random Forest; XGBoost, eXtreme Gradient Boosting; KNN, K-Nearest Neighbors; SVM, Support Vector Machine; LR, Logistic Regression; ACC, accuracy; SEN, sensitivity; SPE, specificity; PPV, positive predictive value; NPV, negative predictive value.

Table S5. The performance of five ML models in Task 2.

Model	AUC (95% CI)	ACC	SEN	SPE	PPV	NPV
RF	0.77 (0.73-0.81)	0.69	0.68	0.70	0.70	0.69
XGBoost	0.73 (0.70-0.77)	0.66	0.62	0.69	0.68	0.65
KNN	0.70 (0.66-0.73)	0.63	0.58	0.69	0.65	0.62
SVM	0.67 (0.64-0.71)	0.62	0.60	0.64	0.62	0.62
LR	0.64 (0.61-0.68)	0.60	0.56	0.64	0.61	0.60

AUC, area under the curve; CI, confidential interval; RF, Random Forest; XGBoost, eXtreme Gradient Boosting; KNN, K-Nearest Neighbors; SVM, Support Vector Machine; LR, Logistic Regression; ACC, accuracy; SEN, sensitivity; SPE, specificity; PPV, positive predictive value; NPV, negative predictive value.

Table S6. AUC of models with different number of features and their comparison in Task 1.

Number of Features	AUC	*p* Value*
11	0.790	-
10	0.787	0.890
9	0.791	0.992
8	0.786	0.868
7	0.788	0.916
6	0.775	0.526
5	0.774	0.505
4	0.739	0.041
3	0.657	<0.001
2	0.648	<0.001
1	0.561	<0.001

**p* values calculated by the Delong test for comparisons of the AUC at each feature reduction step with the AUC of the full feature set. AUC, area under the curve.

Table S7. AUC of models with different number of features and their comparison in Task 2.

Number of Features	AUC	*p* Value*
11	0.735	-
10	0.732	0.790
9	0.734	0.853
8	0.728	0.711
7	0.733	0.837
6	0.709	0.330
5	0.699	0.202
4	0.696	0.177
3	0.710	0.356
2	0.661	0.016
1	0.552	<0.001

**p* values calculated by the Delong test for comparisons of the AUC at each feature reduction step with the AUC of the full feature set. AUC, area under the curve.

Table S8. Univariable and multivariable Cox regression analyses of disease-free survival for Task 1 in the internal test set.

**Variants**	**Univariate**		**Multivariate**	
	**HR (95%CI)**	***p*-Value**	**HR (95%CI)**	***p*-Value**
Age (y)	1.007 (0.972-1.043)	0.699		
Menopausal status (No vs. Yes)	2.663 (1.010-7.022)	**0.048**	2.784 (1.040-7.450)	**0.041**
Tumor location (L vs. R)	1.497 (0.690-3.250)	0.308		
Histologic type (Other vs. NST)	1.154 (0.273-4.876)	0.846		
ER (Negative vs. Positive)	0.608 (0.274-1.346)	0.220		
PR (Negative vs. Positive)	0.985 (0.443-2.189)	0.970		
Ki67 (Low vs. High)	1.478 (0.624-3.501)	0.374		
Model score	2.188 (1.030-4.649)	**0.042**	2.263 (1.058-4.838)	**0.035**

Note—For categorical variables, the first level listed in parentheses in column 1 represents the reference category, and the second level represents the category tested in the model. Only variables with $P < .10$ in univariable analysis were entered into the multivariable model. HR, hazard ratio; CI, confidence interval; L, left; R, right; NST, invasive carcinoma of no special type; ER, estrogen receptor; PR, progesterone receptor.

Table S9. Univariable and multivariable Cox regression analyses of disease-free survival for Task 2 in the internal test set.

**Variants**	**Univariate**		**Multivariate**	
	**HR (95%CI)**	***p*-Value**	**HR (95%CI)**	***p*-Value**
Age (y)	1.004 (0.960-1.050)	0.866		
Menopausal status (No vs. Yes)	2.228 (0.634-7.830)	0.211		
Tumor location (L vs. R)	1.082 (0.412-2.846)	0.872		
Histologic type (Other vs. NST)	1.023 (0.233-4.487)	0.976		
ER (Negative vs. Positive)	0.372 (0.128-1.079)	**0.069**	0.401 (0.137-1.167)	0.094
PR (Negative vs. Positive)	0.774 (0.250-2.401)	0.657		
Ki67 (Low vs. High)	1.319 (0.482-3.607)	0.589		
Model score	0.392 (0.151-1.019)	**0.055**	2.423 (0.930-6.317)	0.070

Note—For categorical variables, the first level listed in parentheses in column 1 represents the reference category, and the second level represents the category tested in the model. Only variables with $P < .10$ in univariable analysis were entered into the multivariable model. HR, hazard ratio; CI, confidence interval; L, left; R, right; NST, invasive carcinoma of no special type; ER, estrogen receptor; PR, progesterone receptor.

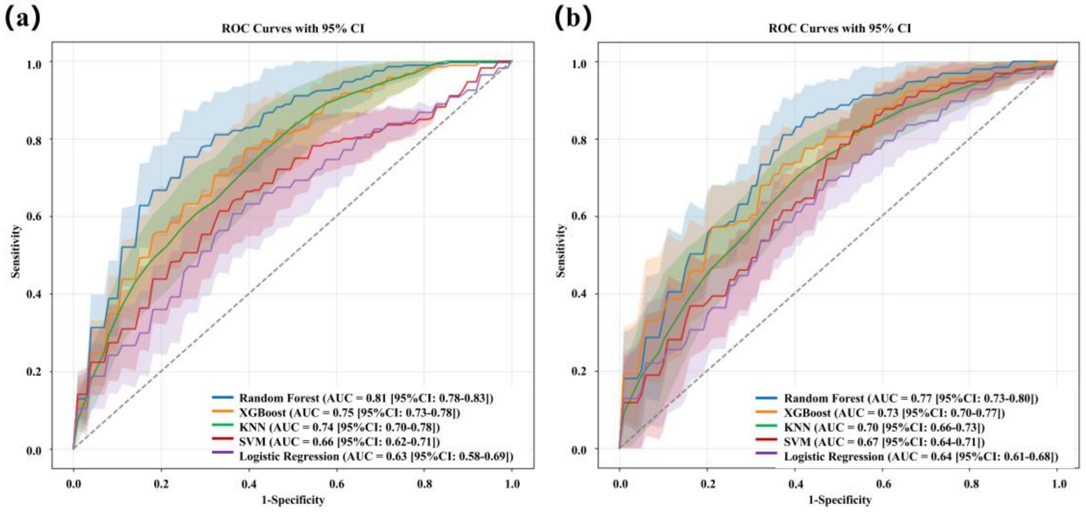

Figure S1. ROC curves for the five machine learning models in Task 1 (**a**) and Task 2 (**b**). CI: confidential index; XGBoost, eXtreme Gradient Boosting; KNN, K-Nearest Neighbors; SVM, Support Vector Machine.

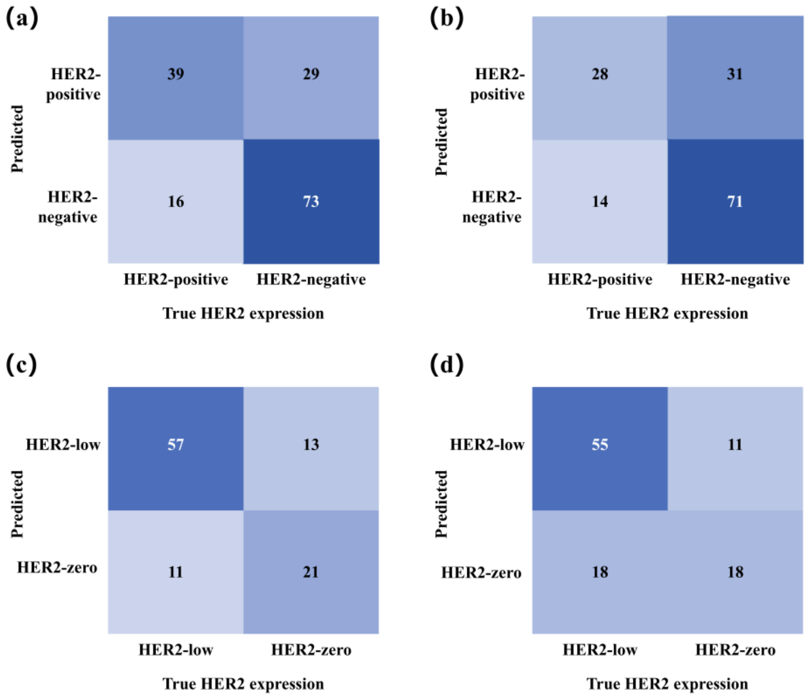

Figure S2. Confusion matrices for model performance on Task 1 and Task 2. (**a-b**) Confusion matrices for Task 1 (a: internal test set, b: external test set), showing the model's performance in predicting HER2-positive versus HER2-negative expressions. (**c-d**) Confusion matrices for Task 2 (c: internal test set, d: external test set), illustrating the model's predictions for HER2-low versus HER2-zero expressions.

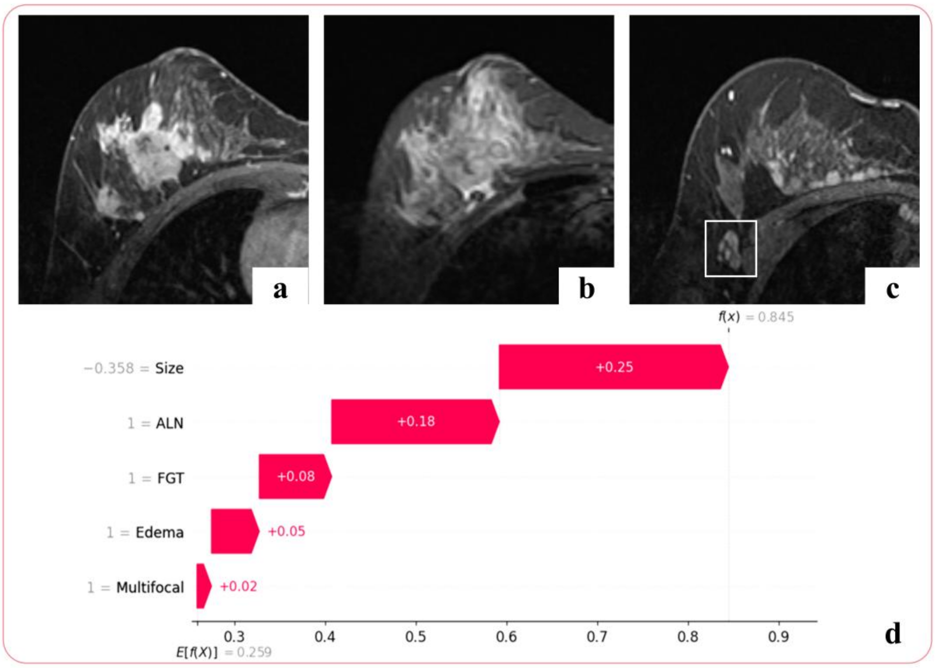

Figure S3. Patient-specific local interpretation of the models for both tasks visualized using SHAP waterfall plots. The case presented involves a patient diagnosed with invasive breast cancer with HER2-positive expression. **(a)** depicts an axial DCE-MRI image highlighting a multiple lesions; **(b)** shows an axial T2WI revealing the presence of peritumoral edema; **(c)** indicates the presence of abnormal ALNs with a heterogeneous FGT. The Task 1 model predicts a 84.5% probability of HER2 positivity for this patient **(d)**.

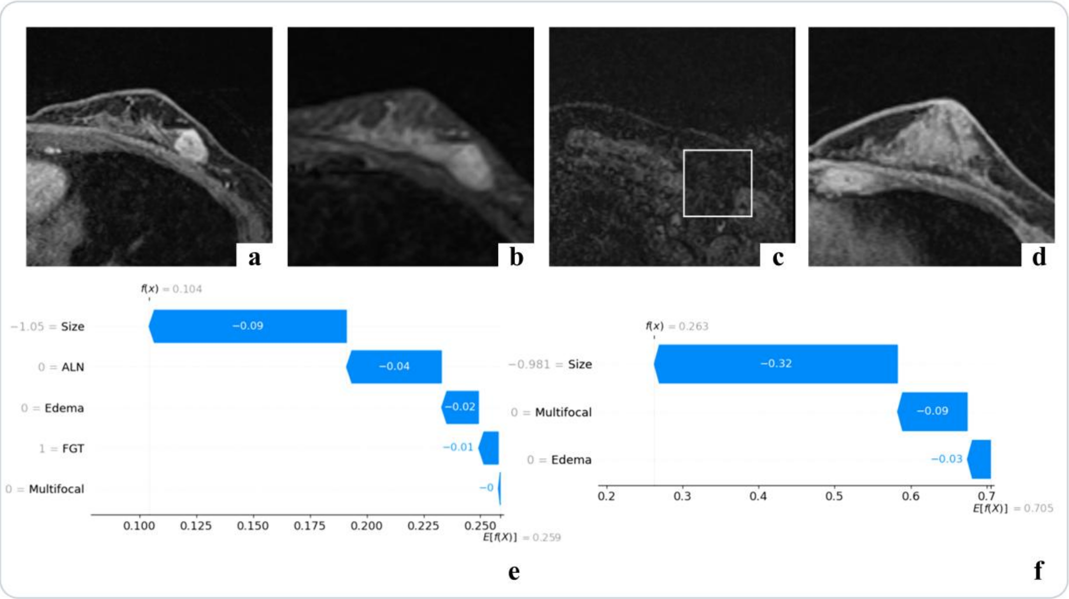

Figure S4. Patient-specific local interpretation of the models for both tasks visualized using SHAP waterfall plots. The case presented involves a patient diagnosed with invasive breast cancer with HER2-zero expression. **(a)** depicts an axial DCE-MRI image highlighting a single lesion; **(b)** shows an axial T2WI revealing the absence of peritumoral edema; **(c)** indicates the absence of abnormal ALNs; and **(d)** demonstrates a heterogeneous/extremely dense FGT. The Task 1 model predicts a 10.4% probability of HER2 positivity for this patient **(e)**, while the Task 2 model outputs a 72.7% probability of HER2 zero expression **(f)**.
